# Extended varenicline treatment in a severe cardiopathic cigarette smoker: a case report

**DOI:** 10.1186/1752-1947-9-29

**Published:** 2015-02-13

**Authors:** Elena Munarini, Chiara Marabelli, Paolo Pozzi, Roberto Boffi

**Affiliations:** Tobacco Control Unit, Fondazione IRCCS Istituto Nazionale dei Tumori, Via Venezian, 1, 20133 Milan, Italy

**Keywords:** Cardiovascular diseases, Side effects, Smoking cessation, Varenicline

## Abstract

**Introduction:**

Tobacco smoking is the leading cause of cardiovascular morbidity and mortality and quitting tobacco use should be fundamental for cardiovascular patients. Varenicline is a smoking cessation pharmacological therapy able to improve the possibilities to successfully achieve this result. In 2011 the US Food and Drug Administration issued a safety announcement that varenicline may be associated with an increased risk of certain cardiovascular adverse events in patients who have cardiovascular disease. Following studies found no significant increase in cardiovascular serious adverse events associated with varenicline. For the first time in the literature, we describe the case of a cardiopathic hard smoker who received varenicline for 9 months without any side effect. By describing this case we want to underline the safety of varenicline, to illustrate the setting and the method that we used to support him and to underline the importance of promoting smoking cessation in heart patients.

**Case presentation:**

Varenicline was used to promote smoking cessation in a 52-year-old Caucasian man who smoked 40 cigarettes per day, despite two ischemic cardiovascular events. He asked for a consultation in a pharmacy’s smoking cessation service and after the assessment phase varenicline was prescribed. Due to his difficulty to quit smoking and given his good tolerance of the drug, we extended the treatment with varenicline to 9 months in order to achieve and maintain a complete smoking abstinence; intensive behavioural counselling was combined with the pharmacological therapy. By using exhaled carbon monoxide measurement we assessed smoking abstinence up to 2 years.

**Conclusions:**

The use of varenicline for a period longer than 6 months has not been described in the literature, particularly in heart patients. The extended varenicline therapy was clinically monitored and allowed the patient to consolidate his abstinence; the intensive behavioural counselling helped him to overcome his strong psychological dependence. Promoting smoking cessation in people who have cardiovascular disease is crucial. Currently available medications, such as varenicline, increase the chances of success and the risk of possible side effects is outweighed by the lifetime benefits and we hope that clinicians use them more frequently and confidently.

## Introduction

Tobacco smoking is the single most preventable cause of disease and death in the world. In many countries, in fact, the prevalence of tobacco consumption is still very high: in Central and Eastern Europe smoking affects more than one-third of adults [1]. At the same time, smoking is the leading cause of cardiovascular (CV) morbidity and mortality: 10 cigarettes per day increase CV mortality in men (18%) and women (31%) [2]. Despite the improvements in treatment of CV diseases the effects of smoking on the CV system remain highly relevant for these patients [3].

Smoking cessation (SC) can be achieved through several ways. According to the International smoking cessation guidelines the use of medications such as nicotine replacement therapy, bupropion or varenicline, coupled with an individual or group counselling are the most effective way to promote continuative SC [4]. Recently, a Cochrane Systematic Review concluded that varenicline improved the likelihood of successfully quitting smoking by two to three-fold relative to pharmacologically unassisted attempts [5] and some studies showed that varenicline may be more efficacious than bupropion in promoting SC [6, 7].

Varenicline is a nicotinic receptor partial agonist and it stimulates nicotine receptors more weakly than nicotine itself does. As a partial agonist it both reduces cravings for and decreases the pleasurable effects of cigarettes and other tobacco products. Through these mechanisms it can assist smokers who want to quit. The summary of product characteristics states that smokers should set a date to stop smoking and treatment with varenicline should start 1 to 2 weeks before this date. The usual starting dose of varenicline is 0.5mg once daily for the first 3 days, then 0.5mg twice daily for the next 4 days, then 0.5mg is continued twice daily or increased to 1mg twice daily thereafter. The maximum dose of varenicline is 1mg twice daily. Varenicline should be taken with a full glass of water, after eating.

Nausea is a side effect that occurs commonly in people taking varenicline. Other less common side effects include headache, difficulty sleeping, and abnormal dreams. Rare side effects reported by people taking varenicline compared to placebo include change in taste, vomiting, abdominal pain, flatulence, and constipation. Serious side effects related to varenicline are uncommon and very serious side effects are rare and they include burning feeling in feet/toes, unusual pain in the legs when walking, chest/jaw/left arm pain, weakness on one side of the body, severe headache, vision changes, confusion, slurred speech, and seizure.

The US Food and Drug Administration (FDA) has approved varenicline use for 12 weeks. If SC has been achieved its administration may be continued for another 12 weeks to maintain abstinence [8]. On June 2011, the FDA issued a safety announcement that varenicline may be associated with a low increased risk of certain CV adverse events in patients who have CV disease [9]; FDA reviewed a randomized clinical trial of 700 smokers with CV disease who were treated with varenicline or placebo. In this trial, varenicline was effective in helping patients quit smoking and remain abstinent from smoking for as long as 1 year. CV adverse events were infrequent overall; however, certain events, including heart attack, angina pectoris, nonfatal myocardial infarction, need for coronary revascularization, and new diagnosis of peripheral vascular disease or admission for a procedure for the treatment of peripheral vascular disease, were reported more frequently in patients treated with varenicline than in patients treated with placebo, but this was not statistically significant. Subsequently a review published in the *Canadian Medical Association Journal* found varenicline to increase the risk of serious adverse CV events, when compared with placebo [10]. So, the FDA required the manufacturer of the drug, Pfizer, to conduct a meta-analysis to further evaluate the CV safety of the drug: it incorporated data from 7002 patients (4190 varenicline and 2812 placebo) that were enrolled in 15 Pfizer-sponsored, randomized, double-blind, placebo-controlled clinical trials of ≥12 weeks treatment duration; findings of CV risk are similar to the findings in the SC clinical trial of patients with stable CV disease that was described in the FDA’s June 2011 announcement.

Finally, a meta-analysis published by the *British Medical Journal* in 2012 that included all double-blind randomized controlled trials of varenicline versus placebo (including eight trials with no events among 1596 participants), focusing on the CV events occurring during drug exposure or within 30 days after its discontinuation, found no significant increase in CV serious adverse events associated with varenicline in over 22 independent trials with 9232 participants [11].

The case that we report here was followed in the particular setting of a pharmacy-based SC service. This service was activated in November 2010 by five communal pharmacies in Milan, Italy [12]; in these pharmacies, smokers’ assistance is provided by both a pharmacist trained on SC and a specialized psychologist of the Tobacco Control Unit from the Fondazione IRCCS Istituto Nazionale dei Tumori (INT). At the first session the Fagerström Test of Nicotine Dependence and Mondor Motivational Test are administered and exhaled carbon monoxide (CO) is measured [13–15]; then, a first-line SC drug is prescribed and the counselling sessions are arranged; when varenicline or bupropion are needed, an INT respiratory physician is available for medical evaluation.

We decided to write this report because it summarizes a lot of concerns about SC: the importance to achieve smoking abstinence in patients with cardiopathic comorbidity, the safety of varenicline even when it is used for an extended period in a cardiopathic patient, the need of SC services able to accept and support smokers and, when if necessary, intensively follow them up in the long term.

## Case presentation

In September 2011 a Caucasian 52-year-old man asked for a consultation in one of the five pharmacies offering SC service in Milan.

He is married with two children; his wife and children were also cigarette smokers.

From the age of 37 he had arterial hypertension. In March 2003 he had an ischemic heart disease in the diagonal branch of his anterior interventricular artery and it was treated with plain old balloon angioplasty (POBA) and one drug-eluting stent application; in July 2011 he had an intrastent restenosis and he underwent a second angioplasty (POBA). He had no history of arrhythmia.

Despite his CV events he has been a continuative heavy cigarette smoker. He started smoking 45 cigarettes per day when he was 20 for a cumulative exposure of 72 pack years. After the first CV event he remained abstinent from smoking just for the time of the hospitalization (4 days). After the second heart attack he quit smoking for 45 days without any pharmacological or psychological help, he then relapsed; although he tried to contain the number of cigarettes up to 8 cigarettes per day until he sought the SC service.

On the first counselling session in the pharmacy, his Fagerström test score was 9 points (revealing high physical dependence), Mondor Questionnaire was 11 (moderate level of motivation to quit) and exhaled CO was 14ppm. An INT physician visited him and registered the following parameters: height 1.75m and weight 85kg; blood pressure was 135/80 with a heart rate of 72 beats per minute; he did not have chronic obstructive pulmonary disease and he was under pharmacological therapy with amlodipine, ramipril and acetylsalicylic acid. The physician found no contraindication to the varenicline treatment; the results of the patient’s blood tests and renal function were normal.

On 22 September 2011 he started varenicline course and he quit smoking on the 14th day, but he relapsed after 5 days reporting to smoke three cigarettes per day during the next 4 months under treatment.

During this period he regularly underwent a psychological counselling session every 15 days. During these sessions, the counsellor tried to motivate him to quit definitively and to overcome his impediments and measured exhaled CO (in this period it was always low level, up to 4ppm); he listed three difficulties that prevented him from quitting: stress at work, the temptation due to living with three cigarette smokers at home, and his refusal to consider himself as a cardiopathic who could not smoke anymore.

On the 20th week after the therapy initiation, he finally quit smoking (exhaled CO=1ppm), even if he reported frequent and intense craving episodes; consequently, due to the absence of varenicline side effects, it was decided to continue the treatment for another 3 months and the frequency of counselling sessions was gradually reduced. At week 24 his creatinine levels were still normal. At the end of the 36th week, he stopped the varenicline treatment.

After 1 year of smoking abstinence, on February 2012, he received the ex-smoking status certificate (exhaled CO=2ppm); during these first 12 months he had a weight gain of 9kg. After 2 years, on February 2013, he was abstinent (exhaled CO=1ppm) and he had lost 8kg through a hypocaloric diet and increased physical activity.

## Conclusions

We report here for the first time in the literature the case of a cigarette smoker who had a serious CV disease who received the first-line SC drug varenicline for a period even longer than recommended, with no CV adverse event. His smoking profile (that is, the presence of a smoking-related illness as well as a high nicotine dependence) indicated to the Tobacco Control team the use of strong pharmacological support plus high-intensity SC counselling; it was decided therefore to prescribe varenicline, despite CV alert [9, 10], as long as no direct experimental evidence suggested that this pharmacological therapy may effectively result in a CV effect [16].

Moreover, even if the varenicline treatment is usually recommended for no longer than 24 weeks, in this particular case we extended the duration of the treatment up to 36 weeks. This choice was led by the conviction that SC was the most important objective to achieve in this patient, due to the severity of his CV illness; furthermore, his psychological process to reach a strong motivation to maintain SC was very slow and he needed a further period of pharmacological support to consolidate his achievement.

Two important factors contributed to this decision: the absence of reported side effects in the first 24 weeks of treatment and the possibility to strictly follow up the patient, as long as the pharmacy SC service was available. In fact, although this patient had a serious CV disease, he had never been involved in any SC program; the open and free access to a SC service allowed him to feel intensively supported in his decision to quit smoking and allowed the Tobacco Control team to pursue his therapy in a safe way. At the same time it must be said that this case required the support team to spend a lot of time and we know it is not easy in the daily practice to have this availability.

Promoting SC in people who have CV disease carries enormous benefits [17] and the potential risks related to the SC drugs are minimal if compared to the lifetime benefits that derive from a successful quit attempt in this subgroup of patients [18].

Too often cardiopathic patients are only invited to quit smoking, but they are not prescribed drugs or SC counselling [19]. The medical staff involved in the management of cardiopathic patients should try to promote SC as the most important preventive and therapeutic decision, weighing the risks of these therapies against the benefits of their use. In relation to varenicline the FDA declares “Health care professionals are advised to weigh the risks of varenicline against the benefits of its use. It is important to note that smoking is a major risk factor for CV disease, and varenicline is effective in helping patients to quit smoking and abstain from it for as long as one year. The health benefits of quitting smoking are immediate and substantial” [20].

## Consent

Written informed consent was obtained from the patient for publication of this case report. A copy of the written consent is available for review by the Editor-in-Chief of this journal.

## Abbreviations

CO: Carbon monoxide
CV: Cardiovascular
FDA: US Food and Drug Administration
INT: Istituto Nazionale dei Tumori
POBA: Plain old balloon angioplasty
SC: Smoking cessation.
